# The Health Food Co.

**Published:** 1877-08

**Authors:** 


### THE HEALTH FOOD COMPANY.

Since the removal of this institution to the store on the north-west corner of Fourth Avenue and Tenth St., opposite the upper end of Stewart's, they appear to enjoy a largely increased trade. Their scientifically prepared foods for infants, for dyspeptics, for diabetics, and for consumptives, are in great demand and seem to give unbounded satisfaction. Their COLD-BLAST WHOLE WHEAT FLOUR has proved the most palatable and nourishing of all flours made from wheat, while the WHITE WHEAT GLUTEN, of which they are the only manufacturers in America, is accomplishing a very remarkable work among a large class of exhausted brains and worn out nervous systems. Their foods are concentrated by the exclusion of all that is inert and the retention of all that is bland, soluble, and nutritive. To concentrate below this, they recognize as disorganization, a thing to be carefully avoided. Our friends who seek to know what to eat in order to be well should address THE HEALTH FOOD COMPANY.

